# PReS-FINAL-2144: Bone mineral status in patients with juvenile idiopathic arthritis after 12 months of treatment with etanercept

**DOI:** 10.1186/1546-0096-11-S2-P156

**Published:** 2013-12-05

**Authors:** G Susic, G Radunovic, N Damjanov, D Novakovic, N Djurovic, R Stojanovic

**Affiliations:** 1 Institute of Rheumatology, Belgrade, Serbia; 2 University of Belgrade, School of Medicine, Belgrade, Serbia

## Introduction

Juvenile idiopathic arthritis (JIA) is a chronic inflammatory disease of childhood. It is associated with decreased bone mineral density (BMD), as result of disturbance of bone metabolism which develops gradually and progressively influenced by many factors, presumably by disease severity.

## Objectives

To examine bone mineral status in patients with JIA after 12 months of treatment with etanercept (ETN).

## Methods

In prospective study of 63 polyarticular JIA pts (46 F, 17 M), median age 15 yrs. Treated with ETN 0,4 mg/kg/2× week. BMD and bone mineral content (BMC) by dual x-ray absorptiometry on the lumbar spine (L2-L4) were assessed. For statistical analysis Z score and bmd_vol _were taken as well. ACR pedi 50 criteria (physician global assessment-PGA, parent's/patient's global assessment, No of joints with limited range of motion-LOM, No of joints with active arthritis, functional ability assessment using CHAQ-Serbian version and ESR) were done at the baseline and one year later.

## Results

We found significant improvement in all six variables represented disease activity, after 12 mo of treatment with ETN (PGA 80%, parent's/patient's global assessment 56%, No of LOM joints 68%, No joints with active arthritis 80%, functional ability 60%, ESR 37% (p < 0.001). At the last visit 54 (85,7%) pts met the ACR pedi 50 criteria, assigned as responders (Group I), while 9 (14,3%) were nonresponders (Group II). After one year of treatment with ETN it was shown high statistically significant increment in all osteodensitometry variables (p < 0.001). Annual enhancement for BMC was 11.7%, for BMD 6.1%, for bmd_vol _3.4% for whole group. Z score improved from -1.03 to -0.81 SD at the last visit. Comparing annually difference of BMD between responders and nonresponders we found statistically significant improvement in the first group (6.78% vs. 2.23%, p < 0.02) as well bmd_vol _(4.31% vs. -0.87%, p < 0.013).

## Conclusion

Our results confirmed significant suppression of disease activity and improvement of bone mineral status in children with JIA after a year of treatment with ETN. Thus reasonable therapeutic approach in preventing osteoporosis in the later life are the new therapeutic options as ETN that could has protective role at structural bone damage.

## Disclosure of interest

None declared.

